# Linkages of NANDA International, NIC and NOC for People Living with HIV Over 50

**DOI:** 10.1093/geroni/igaf122.4154

**Published:** 2025-12-31

**Authors:** Juh Shin, Cheryl Wagner, Elizabeth Swanson, Mary Clarke, Sue Moorhead, Karen Dunn Lopez, Hritik Majgaonkar, Nidhi Naidu

**Affiliations:** George Washington University, Ashburn, Virginia, United States; University of Iowa College of Nursing, Iowa City, Iowa, United States; University of Iowa Center of Nursing Classification and Clinical Effectiveness, Iowa City, Iowa, Iowa, United States; Healthlinx, Davenport, Iowa, United States; University of Iowa, Iowa City, Iowa, United States; University of Iowa, Iowa City, Iowa, United States; George Washington University, Washington, DC, District of Columbia, United States; Geroge Washington University, Arlington, Virginia, United States

## Abstract

Despite advancements in HIV treatment, nursing care for older adults living with HIV (OPLWH) remains insufficiently structured to address their complex, age-related needs. The purpose was to develop linkages between NANDA International, Nursing Outcomes Classification, and Nursing Interventions Classification for older adults living with HIV aged over 50. Data were collected from 16 experienced nurses specializing HIV/AIDS care, who evaluated the relevance of 45 NANDA International, 79 Nursing Outcomes Classification, and 82 Nursing Interventions Classification specific to older adults living with HIV. A table was developed to present frequently used 16 NANDA-I diagnoses in the as critical (relevance ratio ≥0.80) or supplemental (0.60–0.79). The Nursing Outcomes Classifications were linked according to the core components of the selected NANDA-I, followed by Nursing Interventions Classification. Finally, we achieved consensus among experts based on clinical judgment, experience, and established NOC/NIC knowledge. Sixteen core NANDA International were validated as critical, including (i) Risk for Excessive Loneliness linked with Loneliness Severity and Emotional Support; (ii) Risk for Infection linked with Infection Severity and Infection Control; (iii) Inadequate Nutritional Intake linked with Nutritional Status: Nutrient Intake and Nutrition Management; (iv) Impaired Tissue Integrity linked with Tissue Integrity: Skin and Mucous Membranes and Wound Care; and (v) Impaired Memory linked with Memory and Cognitive Stimulation. The validated linkages provide a structured, evidence-based framework for nursing care planning for older adults living with human immunodeficiency virus, emphasizing the integration of psychosocial and physiological health needs. This linkage system supports holistic, individualized nursing care.

